# Multi-centre discriminating concentration determination of broflanilide and potential for cross-resistance to other public health insecticides in *Anopheles* vector populations

**DOI:** 10.1038/s41598-022-26990-6

**Published:** 2022-12-26

**Authors:** Natalie M. Portwood, Magreth F. Shayo, Patrick K. Tungu, Njelembo J. Mbewe, George Mlay, Graham Small, Janneke Snetselaar, Mojca Kristan, Prisca Levy, Thomas Walker, Matthew J. Kirby, William Kisinza, Franklin W. Mosha, Mark Rowland, Louisa A. Messenger

**Affiliations:** 1 Department of Disease Control, Faculty of Infectious Tropical Diseases, London School of Hygiene and Tropical Medicine, London, WC1E 7HT UK; 2 Kilimanjaro Christian Medical University College, Pan African Malaria Vector Research Consortium, Moshi, Tanzania; 3 National Institute for Medical Research, Amani Research Centre, Muheza, Tanzania; 4 Innovative Vector Control Consortium, Liverpool, UK; 5 School of Life Sciences, Gibbet Hill Campus, University of Warwick, Coventry, CV4 7AL UK; 6 PMI VectorLink Project, Abt Associates, 6130 Executive Blvd., Rockville, MD 20852 USA; 7 Department of Environmental and Occupational Health, School of Public Health, University of Nevada, Las Vegas, NV USA

**Keywords:** Genetics, Zoology, Environmental sciences

## Abstract

Novel insecticides are urgently needed to control insecticide-resistant populations of *Anopheles* malaria vectors. Broflanilide acts as a non-competitive antagonist of the gamma-aminobutyric acid receptor and has shown prolonged effectiveness as an indoor residual spraying product (VECTRON T500) in experimental hut trials against pyrethroid-resistant vector populations. This multi-centre study expanded upon initial discriminating concentration testing of broflanilide, using six *Anopheles* insectary colonies (*An. gambiae* Kisumu KCMUCo, *An. gambiae* Kisumu NIMR, *An. arabiensis* KGB, *An. arabiensis* SENN, *An. coluzzii* N’Gousso and *An. stephensi* SK), representing major malaria vector species, to facilitate prospective susceptibility monitoring of this new insecticide; and investigated the potential for cross-resistance to broflanilide via the A296S mutation associated with dieldrin resistance (*rdl*). Across all vector species tested, the discriminating concentration for broflanilide ranged between LC_99_ × 2 = 1.126–54.00 μg/ml or LC_95_ × 3 = 0.7437–17.82 μg/ml. Lower concentrations of broflanilide were required to induce complete mortality of *An. arabiensis* SENN (dieldrin-resistant), compared to its susceptible counterpart, *An. arabiensis* KGB, and there was no association between the presence of the *rdl* mechanism of resistance and survival in broflanilide bioassays, demonstrating a lack of cross-resistance to broflanilide. Study findings provide a benchmark for broflanilide susceptibility monitoring as part of ongoing VECTRON T500 community trials in Tanzania and Benin.

## Introduction

The scale-up of key diagnostic, treatment and vector control interventions, particularly long-lasting insecticidal nets (LLINs) and indoor residual spraying (IRS)^1^, has led to a global decline in malaria incidence. Approximately, 1.7 billion malaria cases and 10.6 million malaria deaths were averted between 2000 and 2020^1,2^, with LLINs and IRS accounting for 68% and 10% of these achievements, respectively^2^. Insecticidal products currently used in malaria vector control predominantly target indoor host-seeking or resting *Anopheles* vectors, inducing lethality following exposure. However, widespread deployment of these interventions has placed high levels of selection pressure on mosquito populations, leading to the evolution and spread of insecticide resistance and coincident declines in malaria control^1,3^. Insecticide resistance has become established in malaria vector populations to four classes of insecticides that have been historically used for public health use: pyrethroids, carbamates, organophosphates, and organochlorines^4,5^. Pyrethroid resistance is of particular concern, because until recently, it was the only class of insecticide utilised in LLINs^6^.

In response to the threat of insecticide resistance, substantial investments have been made in the development and repurposing of new insecticides and chemical classes, with novel modes of action, to improve malaria vector control and potentially mitigate further resistance selection. IRS operational strategies in sub-Saharan Africa currently use organophosphate (Actellic 300CS; containing pirimiphos-methyl, an acetylcholinesterase inhibitor) or neonicotinoid products (SumiShield 50WG or Fludora Fusion; containing clothianidin, a nicotinic acetylcholine receptor agonist)^7,8^. Two generations of LLINs containing a pyrethroid insecticide and a second partner chemical, piperonyl butoxide (PBO; an insecticide synergist)^9^, a pyrrole (chlorfenapyr; an oxidative phosphorylation uncoupler)^10^ or an insect growth regulator (pyriproxyfen; a juvenile hormone analogue)^11^, have been evaluated, with strong epidemiological evidence to support the use of PBO-LLINs (Olyset Plus)^12^ and chlorfenapyr-LLINs (Interceptor G2)^13^ to control malaria transmitted by pyrethroid-resistant vector populations. However, reports of incipient resistance to these new insecticides are beginning to emerge^14^, soon after they have been deployed, highlighting the urgent need for additional chemicals, with distinct target sites, to incorporate into effective resistance management strategies^15^.

Broflanilide (tradename TENEBENAL) is a novel insecticide discovered by Mitsui Chemicals Agro, Inc^16^, which has been formulated as a wettable powder for IRS (VECTRON T500). It has a unique chemical structure characterized as a *meta*-diamide, that acts as a non-competitive antagonist (NCA) of the gamma-aminobutyric acid (GABA) receptor of chloride channels in the insect inhibitory nervous system, causing mosquito mortality by hyperexcitation and convulsion^17^. Broflanilide has been classified by the Insecticide Resistance Action Committee (IRAC)^17^ as a GABA-gated chloride channel allosteric modulator (IRAC Group 30). Broflanilide has low mammalian toxicity and a good safety profile; antagonist activities of *meta*-diamides are considerably lower in human GABA_A_Rα1β2γ2S, mammalian GABA_A_Rα1β3γ2S and human GlyR α1β receptors than in insect RDL GABA receptors, due to the presence of A288G in human GlyR α1β^18^. Furthermore, physiochemical data indicate that broflanilide is stable to hydrolysis and soil photolysis, giving it the potential for long-lasting application in IRS but low environmental persistence; it has low solubility in water (0.71 mg/L at 20 °C), its vapor pressure of 6.6 × 10^–11^ torr and Henry’s law Constant of 3.0 × 10–14 atm-m^3^/mol suggest that volatilization is not a major dissipation pathway and finally soil adsorption coefficient (KF) values of 113 to 248 mL/g indicate low mobility in soil. Evaluation of the residual efficacy of VECTRON T500 has been coordinated between the London School of Hygiene and Tropical Medicine (LSHTM), the Kilimanjaro Christian Medical University (KCMUCo), the National Institute for Medical Research (NIMR) in Tanzania, Mitsui Chemical Agro Inc (MCAG) and the Innovative Vector Control Consortium (IVCC). To date, broflanilide has demonstrated effectiveness against pyrethroid-susceptible and -resistant vector populations^19,20^. In the IRS product, VECTRON T500, broflanilide has shown prolonged effectiveness in experimental hut trials in Tanzania and Benin in comparison with World Health Organization (WHO) prequalified products^19,20^. VECTRON T500 is now under evaluation in non-inferiority community trials in both countries to provide data in support of its evaluation by the WHO Prequalification Unit, Vector Control Product Assessment Team (PQT/VCP) as a new IRS product for insecticide resistance management^21^.

Insecticides targeting the GABA-gated chloride receptors have been highly effective, being used extensively across Africa in the 1960s–1970s^4^, for agriculture and public health, including cyclodienes (dieldrin), phenyl pyrazoles (fipronil) and isoxazolines (fluralaner)^22^. However, the 2001 Stockholm Convention on Persistent Organic Pollutants prohibited the use of cyclodienes due to their slow degradation and environmental persistence^23^. Despite this ban, there is evidence indicating that the mutations conferring resistance to dieldrin (*rdl*) have persisted decades later in malaria vector populations^24,25^. Whilst some in silico studies have demonstrated that the mode of action of broflanilide is distinct from other NCAs targeting the GABA-gated chlorine channel, including dieldrin, fipronil, lindane and α-endosulfan^26,27^, little biological evidence has been generated to date with mosquito strains, to establish whether there is any cross-resistance to broflanilide via the mutation in the GABA-gated chloride receptor leading to dieldrin resistance (*rdl*)^26–28^.

The aim of this multi-centre study was to expand upon initial discriminating concentration (DC) testing of broflanilide^20,28^, defined as the concentration of insecticide that in a standard period of exposure, is used to discriminate the proportions of susceptible and resistant phenotypes in a sample of a mosquito population^29^, using additional *Anopheles* vector species, to facilitate prospective susceptibility monitoring of this new insecticide; and to investigate the potential of the A296S *rdl* resistance mutation in the GABA receptor gene to offer cross-resistance between broflanilide and dieldrin.

## Results

### Broflanilide discriminating concentration determination

**Figure 1:**
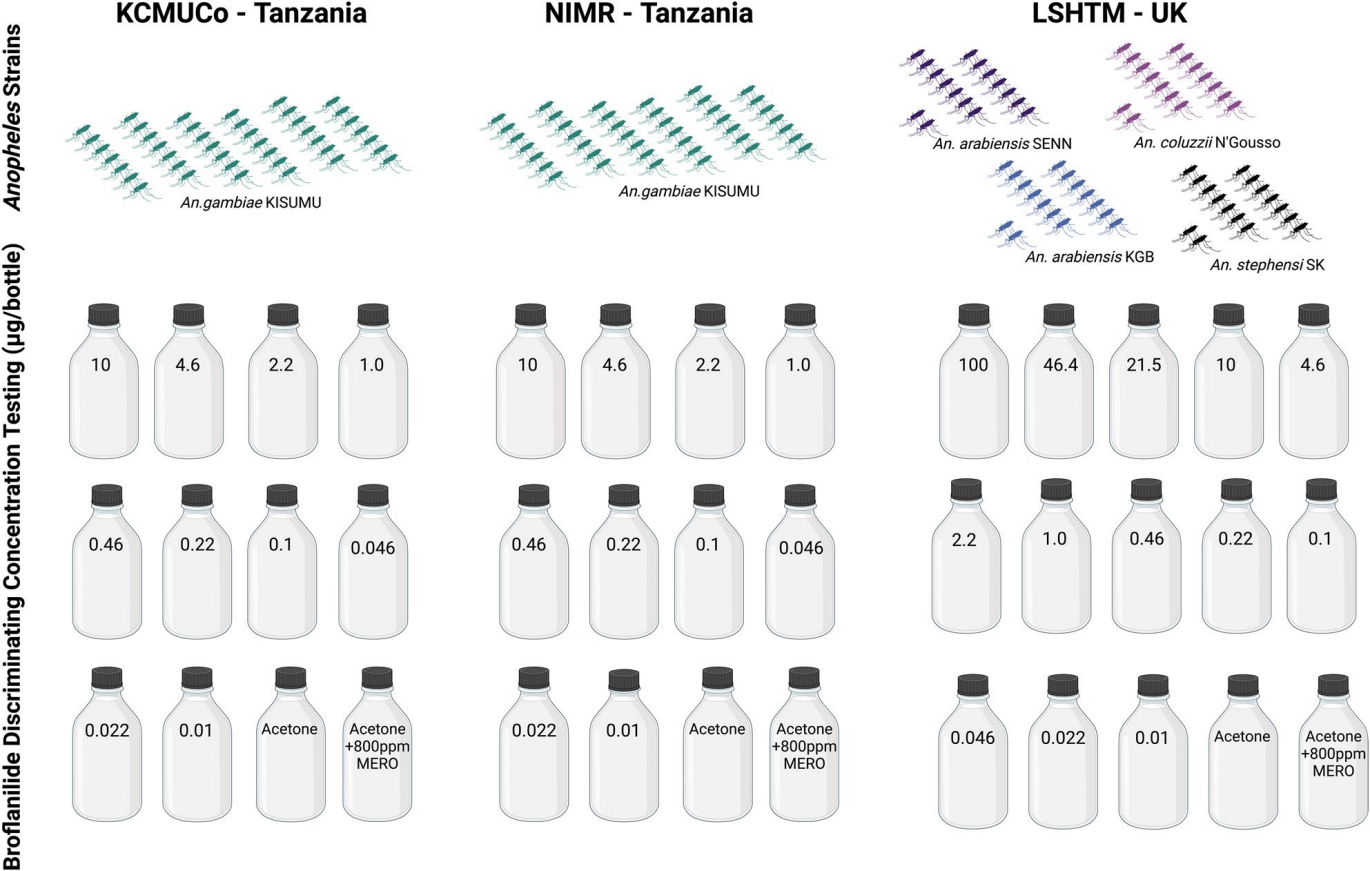
Multi-centre broflanilide discriminating concentration testing study: experimental design. Figure created using BioRender.com.

This multi-centre study tested a total of 7370, 2–5 day old, unfed female *Anopheles* mosquitoes from six colony strains (*An. gambiae* Kisumu KCMUCo, n = 1812; *An. gambiae* Kisumu NIMR, n = 860; *An. coluzzii* N’Gousso, n = 1640; *An. arabiensis* KGB, n = 873; *An. arabiensis* SENN, n = 476; and *An. stephensi* SK, n = 1709), across different concentrations of broflanilide (Fig. 1).

**Figure 2:**
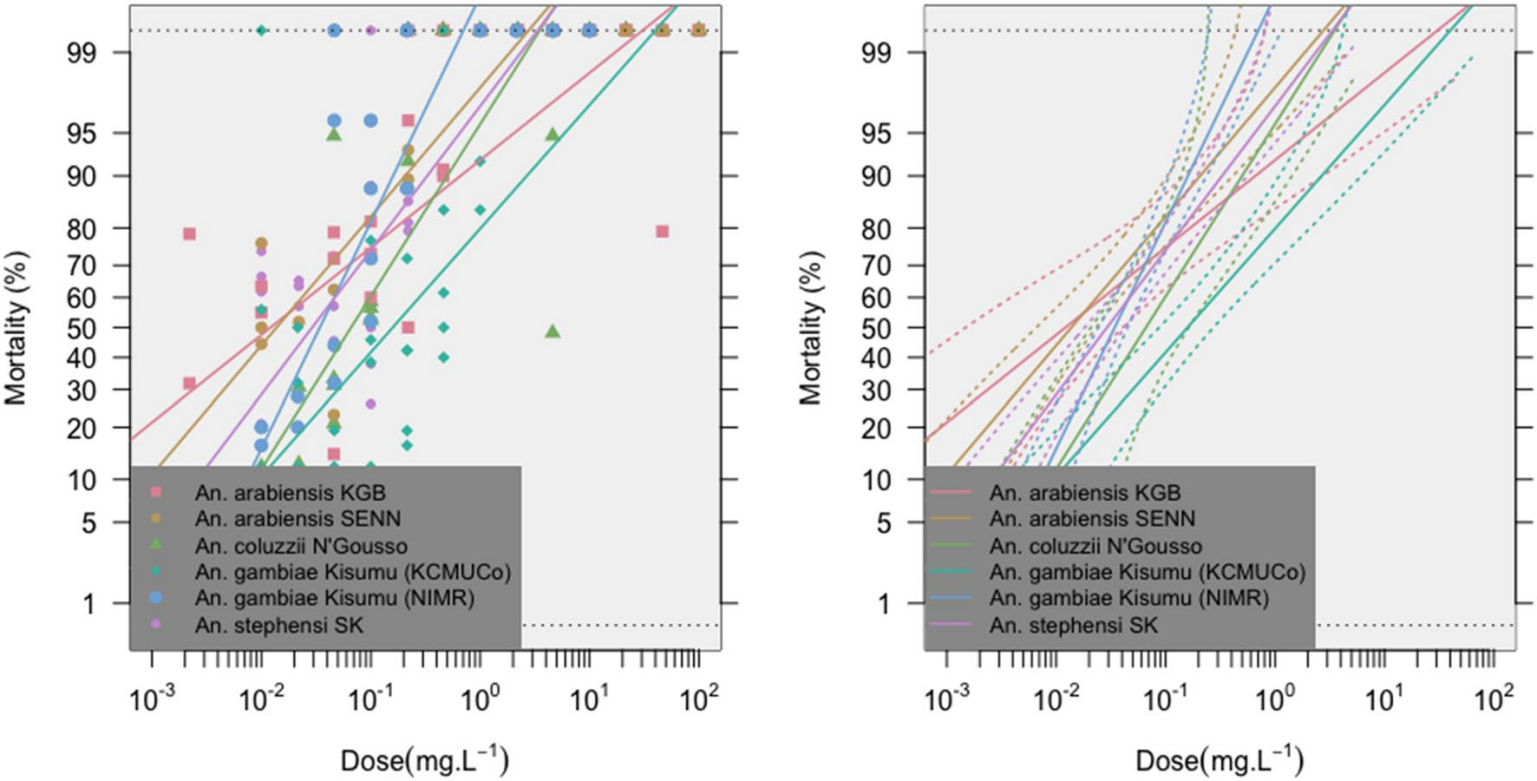
Linear relationships between probit-transformed mortality rates and log-dose of broflanilide for different *Anopheles* insectary strains (left), with 95% confidence intervals (right).

A clear mortality-dose response following broflanilide exposure was evident with all insectary strains tested (Fig. 2). Table 1 details lethal doses (%) of broflanilide required for mortality of all six *Anopheles* insectary strains, with corresponding DCs presented in Table 2, calculated according to two methodologies^30,31^. Of the five insecticide-susceptible mosquito strains tested (*An. gambiae* Kisumu KCMUCo, *An. gambiae* Kisumu NIMR, *An. coluzzii* N’Gousso, *An. arabiensis* KGB and *An. stephensi* SK), the lowest DC was observed for *An. gambiae* Kisumu NIMR (LC_99_ × 2 = 1.126 μg/ml [95% CI 0.197–2.78 μg/ml]; LC_95_ × 3 = 0.7437 μg/ml [95% CI 0.0882–2.2338 μg/ml]), followed by *An. stephensi* SK (LC_99_ × 2 = 4.72 μg/ml [95% CI 2.08–8.04 μg/ml]; LC_95_ × 3 = 1.95 μg/ml [95% CI 0.5652–4.17 μg/ml]) (Table 2). By comparison, the highest DC was recorded for *An. gambiae* Kisumu KCMUCo (LC_99_ × 2 = 54.00 μg/ml [95% CI 28.00–134.00 μg/ml]; LC_95_ × 3 = 17.82 μg/ml [95% CI 10.41–33.00 μg/ml]) (Table 2), indicating substantial variation in mortality-dose response between the two insectary colonies derived from the same original stock (*An. gambiae* Kisumu). The insecticide-resistant mosquito strain (dieldrin-resistant: *An. arabiensis* SENN) presented an intermediate DC as determined by the method of Lees et al*.*^31^ and as determined following the WHO approach^32^ (LC_99_ × 2 = 3.76 μg/ml [95% CI 0.92–7.96 μg/ml]; LC_95_ × 3 = 1.33 μg/ml [95% CI 0.1506–4.11 μg/ml]), when compared to the other insectary colonies (Table 2).

**Table 1:** Lethal concentrations (%) of broflanilide (μg/ml) required for mortality of six *Anopheles* insectary strains.

Mosquito colony	LC50 [95% CI]	LC95 [95% CI]	LC99 [95% CI]
*An. gambiae* Kisumu (KCMUCo)	0.1589 [0.0360–0.3858]	5.9400 [3.470–11.00]	27.00 [14.00–67.00]
*An. gambiae* Kisumu (NIMR)	0.0342 [0.0016–0.1657]	0.2479 [0.0294–0.7446]	0.563 [0.0985–1.39]
*An. coluzzii* N’Gousso	0.0661 [0.0129–0.1837]	0.8898 [0.3752–1.57]	2.61 [1.46–3.98]
*An. arabiensis* KGB	0.0122 [0.0003–0.0795]	2.05 [0.5594–4.80]	17.00 [7.57–45.00]
*An. arabiensis* SENN	0.0138 [0.0003–0.0979]	0.4449 [0.0502–1.37]	1.88 [0.46–3.98]
*An. stephensi* SK	0.0291 [0.0029–0.1144]	0.6515 [0.1884–1.39]	2.36 [1.04–4.02]

**Table 2:** Estimated broflanilide discriminating concentrations (μg/ml).

Mosquito Colony	DC (LC_99_ × 2) [95% CI]	DC (LC_95_ × 3) [95% CI]
*An. gambiae* Kisumu (KCMUCo)	54.00 [28.00–134.00]	17.82 [10.41–33.00]
*An. gambiae* Kisumu (NIMR)	1.126 [0.197–2.78]	0.7437 [0.0882–2.2338]
*An. coluzzii* N’Gousso	5.22 [2.92–7.96]	2.67 [1.13–4.71]
*An. arabiensis* KGB	34.00 [15.14–90.00]	6.15 [1.68–14.4]
*An. arabiensis* SENN	3.76 [0.92–7.96]	1.33 [0.1506–4.11]
*An. stephensi* SK	4.72 [2.08–8.04]	1.95 [0.5652–4.17]

Pairwise Bonferroni comparisons indicated significant differences in dose-mortality responses between *An. arabiensis* KGB and *An. gambiae* Kisumu KCMUCo (*p* = 0.00076), *An. arabiensis* SENN and *An. gambiae* Kisumu KCMUCo (*p* = 0.00121), *An. arabiensis* SENN and *An. gambiae* Kisumu NIMR (*p* = 0.00875), *An. stephensi* SK and *An. gambiae* Kisumu KCMUCo (*p* < 0.0001) and *An. gambiae* Kisumu KCMUCo and *An. gambiae* Kisumu NIMR (*p* < 0.0001).

### Dieldrin cross-resistance testing

**Figure 3:**
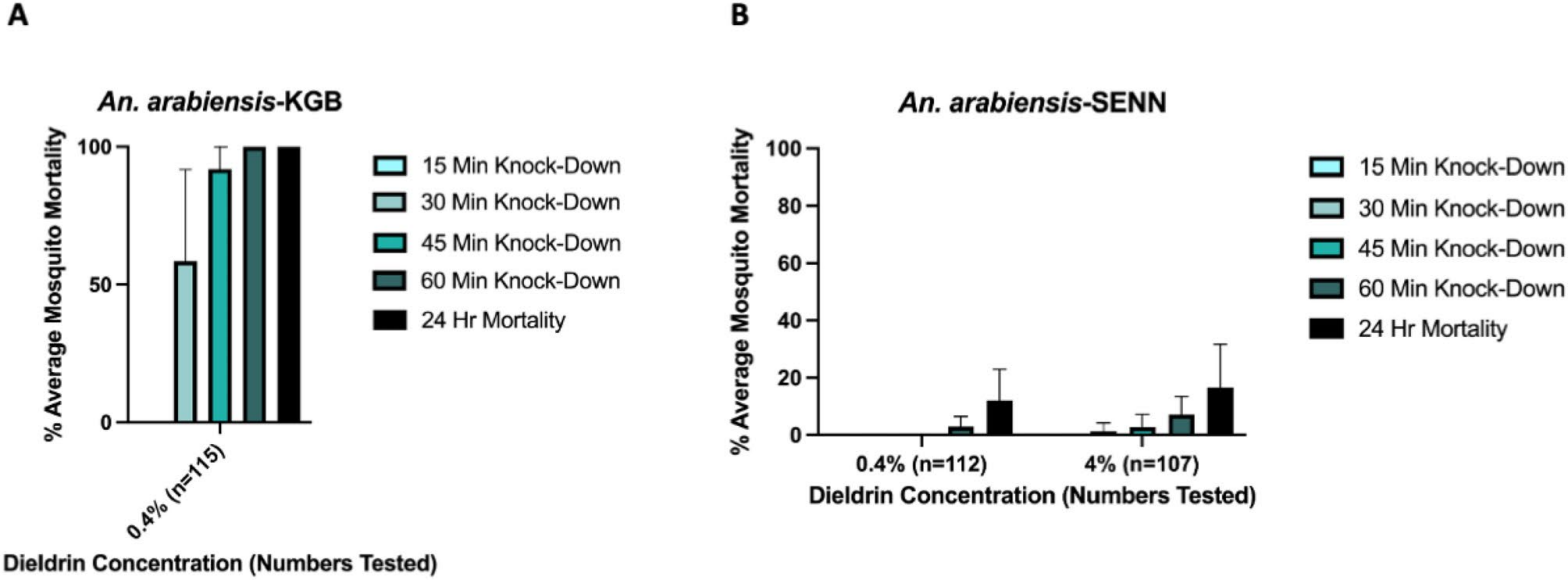
*An. arabiensis* KGB (**A**) and *An. arabiensis* SENN (**B**) mortality after exposure to dieldrin in WHO susceptibility tests. Error bars represent 95% confidence intervals (CIs).

To confirm the resistance profiles of both *An. arabiensis* colonies, initial WHO susceptibility tests were performed on *An. arabiensis* SENN (dieldrin-resistant) and *An. arabiensis* KGB (dieldrin-susceptible). A total of 115 KGB individuals were exposed to the discriminating concentration of dieldrin (0.4%) with 100% mortality observed after 60 min (Fig. 3A); demonstrating that this strain was susceptible to dieldrin.

Average 24-h mortality of 112 *An. arabiensis* SENN tested using 0.4% dieldrin impregnated filter papers was 12.1% (95% CI 1.17%-22.9%) (Fig. 3B). For the 107 *An. arabiensis* SENN mosquitoes tested with 4% dieldrin impregnated filter papers (10X discriminating concentration), average 24-h mortality was 16.5% (95% CI 1.33–31.73) (Fig. 3B); confirming that this strain was highly resistant to dieldrin. All *An. arabiensis* SENN which survived 4% dieldrin exposure possessed the A296S mutation. All *An. arabiensis* SENN tested in dieldrin bioassays were confirmed as *An. arabiensis* by species-specific PCR.

Resistance ratios between *An. arabiensis* SENN (dieldrin-resistant) and *An. arabiensis* KGB (dieldrin-susceptible) were 1.13 [95% CI 0.7879–1.63] at the LC_50_, indicating an absence of cross-resistance between broflanilide and dieldrin. A subset of *An. arabiensis* SENN (n = 290) tested against different broflanilide concentrations were screened for the presence of *rdl* A296S to investigate the potential for this resistance mechanism to mediate cross-resistance against broflanilide. There was no association between *rdl* A296S genotype and survival or death following exposure to any broflanilide concentration (Fisher’s exact test = 0.6019). All *An. arabiensis* SENN screened for *rdl* A296S were confirmed as being *An. arabiensis* by species-specific PCR.

## Discussion

The development of novel insecticide formulations for IRS whose efficacies are not compromised by pre-existing cross-resistance in vector populations, is crucial to sustain current gains in malaria vector control^33^. This multi-centre study builds upon initial broflanilide DC testing performed with single insecticide-susceptible insectary colonies, using six mosquito strains, representing major *Anopheles* species; *An. gambiae* and *An. arabiensis* are sympatric malaria vectors across sub-Saharan Africa^34^, *An. coluzzii* is a pervasive malaria vector species in West Africa^35^ and *An. stephensi* is the primary urban vector species in the Indian subcontinent^36^, which has become an invasive rural species in the Horn of Africa^37^ and has recently been detected in Nigeria^38^. Study results demonstrated significant heterogeneity in mortality-dose responses following broflanilide exposure, between *Anopheles* species (e.g. *An. stephensi* SK *vs*. *An. gambiae* Kisumu KCMUCo), within *Anopheles* species (e.g. *An. arabiensis* KGB *vs. An. arabiensis* SENN) and even between the same insectary strain maintained at different testing facilities (*An. gambiae* Kisumu KCMUCo *vs. An. gambiae* Kisumu NIMR). Across all vector species tested, the ranges of DC generated by this study were 1.126 μg/ml to 54.00 μg/ml (LC_99_ × 2) or 0.7437 μg/ml to 17.82 μg/ml (LC_95_ × 3). These estimates provide an initial benchmark for broflanilide susceptibility monitoring, as part of ongoing VECTRON T500 community trials in Tanzania and Benin^21^. Further studies will be required on different *Anopheles* species and populations in order to identify what will be the definitive DC for broflanilide susceptibility monitoring in conjunction with the use of VECTRON T500 in malaria vector control programmes.

Our findings align with previous studies which estimated the LC_95_ × 3 = 11.91 μg/ml [95% CI 8.253–21.618]^28^ with *An. gambiae* Kisumu LITE and LC_95_ × 3 = 210 μg/ml [95% CI 115.5–423.3]^20^ with *An. gambiae* Kisumu CREC. The difference between the DCs in these studies may be explained by a difference in the method for coating bottles used in bioassay testing. In the current study, technical grade broflanilide was dissolved in acetone with 800 ppm Mero® (81% rapeseed oil methyl ester), as recommended by the commercial manufacturer. Although the role of Mero® in the pickup and uptake of some insecticides is not yet fully understood, it is known that it prevents insecticide crystallization, which can inhibit absorption across the insect cuticle, allowing broflanilide to remain in an amorphous state throughout bioassay testing. The addition of Mero®, therefore, increases the efficacy of broflanilide in bottle bioassays with mosquitoes, i.e., it decreases the concentration of broflanilide needed for lethality. Similarly, the efficacy of clothianidin in bottle bioassays has also been shown to be enhanced by the inclusion of Mero® when coating bottles^39^.

The differences between bioassay results using the same mosquito strain (*An. gambiae* Kisumu) maintained at two separate testing facilities (KCMUCo and NIMR) raises some interesting questions regarding direct comparability of insectary colony data. Differences in mosquito rearing conditions, including larval rearing conditions (e.g. crowding, access to nutrition)^40^, time of testing (e.g. night or day)^41,42^, temperature and humidity^43^, mosquito age^44^ and physiological status^45^ can have a significant effect on observed bioassay mortality. Whilst every effort is made to maintain standardized test conditions, according to WHO protocols^30^, even differences of 4 °C during holding periods can have a significant effect on mosquito mortality^43^, with lower temperatures associated with reduced mortality. Finally, an unascertainable amount of variation between the *An. gambiae* Kisumu strains maintained at different testing facilities may be attributable to long-term genetic divergence, and in turn, differences in relative colony fitness since these mosquito populations have been maintained in separate facilities for more than a decade. These observations support periodic in-depth strain characterization at both phenotypic and genotypic levels, as has been reported for recently colonized insecticide-resistant colonies^46,47^, to strengthen future laboratory screening of new insecticides.

A secondary objective of this study was to investigate whether there was any biological basis for cross-resistance to broflanilide via the A296S mutation in the GABA-gated chloride receptor leading to dieldrin resistance (*rdl*). Despite the ban of dieldrin decades ago, the A296G and A296S *rdl* mutations have persisted in some contemporary vector populations at high frequencies^48–50^. We observed no evidence for cross-resistance to broflanilide in this study supporting its deployment in areas of pre-existing *rdl*; indeed, lower concentrations of broflanilide were required to induce complete mortality of the dieldrin-resistant *An. arabiensis* SENN strain, compared to its susceptible counterpart, *An. arabiensis* KGB, which may in part be explained by the fitness costs associated with highly insecticide-resistant populations, as shown in previous field studies^51,52^. This was further reinforced by a lack of association between the A296S *rdl* mutation and the outcomes of broflanilide bottle bioassays. Three additional amino acids, which surround the broflanilide binding pocket in the GABA receptor, have been identified that can disrupt insecticide binding: G331, I272 and L276^28^. Screening of the Ag1000 genome data has failed to identify any naturally-occurring mutations in these amino acids in *Anopheles* field populations^28^. These genetic regions warrant inclusion in newly developed amplicon-sequencing panels^53^, which are being rolled out to monitor insecticide resistance across *Anopheles* vector populations, in conjunction with standard insecticide susceptibility monitoring.

The variability in mortality-dose response to broflanilide, evidenced in this study, strongly advocates for further broflanilide DC testing, using additional insecticide-susceptible *Anopheles* colonies, particularly an *An. funestus* strain; this vector species predominates across southern sub-Saharan Africa and plays an increasing role in malaria transmission in areas where other vector species have been controlled by insecticidal interventions^54^. Unfortunately, this was not feasible for inclusion in this multi-centre study, due to notorious difficulties rearing this particular species under controlled insectary conditions^55^. Broflanilide testing using wild *Anopheles* populations is also needed to demonstrate the efficacy of this novel insecticide to control pyrethroid-resistant vectors and to assess variability in the tolerance of these populations to broflanilide. Previous laboratory studies have demonstrated a lack of cross-resistance between mechanisms of resistance possessed by pyrethroid-resistant insectary strains and broflanilide^20,28^. However, a plethora of complex coinciding, insecticide resistance mechanisms can be found in natural *Anopheles* populations^56–61^, which are not adequately reflected in genetically homogenous insectary colonies.

## Conclusions

This multi-centre study, using six *Anopheles* insectary colonies, representing major malaria vector species, determined the putative discriminating concentration for broflanilide to range between LC_99_ × 2 = 1.126 to 54.00 μg/ml or LC_95_ × 3 = 0.7437 to 17.82 μg/ml. Comparison of the susceptibility of dieldrin-resistant and -susceptible *An. arabiensis* colonies provided no phenotypic or genotypic evidence for cross-resistance to broflanilide via the A296S *rdl* mutation in the GABA-gated chloride receptor leading to dieldrin resistance. Use of the adjuvant Mero® increased broflanilide efficacy, highlighting the need to standardize bottle bioassay testing for this new insecticide. Differences in bioassay results using the same mosquito strain (*An. gambiae* Kisumu) maintained at two separate facilities raised issues regarding direct comparability of insectary colony data and emphasizes the need for periodic in-depth strain characterization to strengthen future laboratory screening of new insecticides. Our study findings provide a benchmark for broflanilide susceptibility monitoring as part of ongoing VECTRON T500 community trials in Tanzania and Benin.

## Methods

### Mosquito strains

Six *Anopheles* insectary colonies were used for this multi-centre evaluation: susceptible *An. gambiae* Kisumu (KCMUCo and NIMR), susceptible *An. coluzzii* N’Gousso (LSHTM)*,* susceptible *An. stephensi* SK (LSHTM), susceptible *An. arabiensis* KGB (LSHTM) and dieldrin-resistant *An. arabiensis* SENN (LSHTM). *An. gambiae* Kisumu (KCMUCo and NIMR) is a laboratory strain colonised in 1953 from Kenya. Susceptible *An. gambiae* Kisumu at KCMUCo is routinely characterized three to four times in a year, with respect to body weight, wing length and both phenotypic and genotypic resistance profiles. Prior to this study, *An. gambiae* Kisumu KCMUCo was confirmed as susceptible to alpha-cypermethrin (pyrethroid) and bendiocarb (carbamate) in WHO tube tests^62^; species identification was confirmed by PCR^63^ and screening for L1014S, L1014F and G119S-Ace-1 did not detect the presence of any insecticide resistance associated mutations in this colony. Susceptible *An. gambiae* Kisumu at NIMR is routinely characterised every three months, with respect to phenotypic and genotypic resistance profiles. Prior to this study, *An. gambiae* Kisumu NIMR was confirmed as susceptible to deltamethrin and permethrin (pyrethroids) in WHO tube tests^62^; species identification was confirmed by PCR^63^ and screening for L1014S and L1014F did not detect the presence of any insecticide resistance associated mutations in this colony. *An. coluzzii* N’gousso (LSHTM) is a laboratory-strain colonised in 2006 from field mosquitoes collected in Cameroon. CDC bottle bioassays using deltamethrin and permethrin have established this colony as pyrethroid-susceptible^64^; PCR has confirmed species identification and that this colony lacks L1014S and L1014F mutations^65^* . An. stephensi* SK (LSHTM) is a laboratory strain colonised from Pakistan in 1982^66^. CDC bottle bioassays using deltamethrin and permethrin have established that this colony is pyrethroid-susceptible^64^ and species identification is regularly confirmed on the basis of morphological features^67^. *An. arabiensis* KGB (LSHTM) is a laboratory strain colonised in 1975 from Zimbabwe. *An. arabiensis* SENN (LSHTM) is a laboratory strain colonised in 1969 from Sudan^68^. This strain has been exposed to dieldrin and confirmed resistant due to the GABA-gated chloride receptor mutation (Ala296Ser). Further characterization of the latter two strains is described below.

In all three testing facilities, all life-cycle stages of colony mosquito populations were maintained under standard insectary conditions (25–27 °C, 80% relative humidity, light:dark cycles of 12-h each). In LSHTM mosquito larvae were reared in large white trays, with 12-h light–dark cycles, and fed NISHIKOI staple fish food pellets (Nishikoi, UK). In KCMUCo and NIMR, mosquito larvae were reared in large white round bowls, with 12-h light–dark cycles, and fed with TetraMin (Tetra, U.S.).

Adult mosquitoes were kept in cages of ~ 30 × 30 × 30 cm at varying densities, with 10% glucose provided ad libitum. In LSHTM, colony cages were maintained by regular blood feeding using a Hemotek feeder. In KCMUCo and NIMR, colony cages were maintained by regular blood feeding on Guinea Pigs*.*

### Broflanilide discriminating concentration testing

A discriminating concentration is defined as the concentration of insecticide that in a standard period of exposure, is used to discriminate the proportions of susceptible and resistant phenotypes in a sample of a mosquito population^29^. Discriminating concentration testing of broflanilide was undertaken using the CDC bottle bioassay method, but with minor modifications to the published guidelines (Fig. 1)^64^. Probit analysis was used to determine thirteen concentrations of broflanilide for testing (100, 46.4, 21.5, 10, 4.6, 2.2, 1, 0.46, 0.22, 0.1, 0.046, 0.022 and 0.01 μg/ml). Technical grade broflanilide (Mitsui Agro, Inc., Japan) was dissolved in acetone with 800 ppm Mero®; the adjuvant Mero® was used to ensure the insecticide was distributed evenly throughout each bottle and to prevent crystallisation of broflanilide during the conduct of bioassays. Control bottles consisting of acetone alone and acetone + 800 ppm Mero® were run in parallel during each bioassay.

Each Wheaton 250 ml bottle and cap was coated using 1 ml of insecticide solution by rolling it and inverting the bottle. In parallel, control bottles were coated with either 1 ml acetone or 1 ml acetone + 800 ppm Mero® per bottles. Once coated, all bottles were covered with a cotton sheet and left to dry in the dark overnight; and were washed thoroughly and re-coated before every test. During bioassays, replicates of 20–25, two-to-five-day old, unfed female mosquitoes were exposed for 1-h. Mosquito mortality was recorded every 15 min up to 1 h. Surviving mosquitoes were supplied with 10% glucose and held for 72-h, with mortality recorded every 24-h.

### WHO insecticide susceptibility tests

To confirm the resistance profiles of both of the *An. arabiensis* colonies used in this study, WHO susceptibility tests were performed using *An. arabiensis* SENN (dieldrin-resistant) and *An. arabiensis* KGB (dieldrin-susceptible) to measure dieldrin susceptibility, following standard procedures^69^. Replicates of 20–25, two-to-five-day old, unfed female mosquitoes were released into WHO holding tubes. After acclimatization of the mosquitoes for one hour in the vertical position, mosquitoes were blown into exposure tubes containing WHO dieldrin (0.4% and 4%) impregnated filter papers or control papers containing risella oil (Universiti Sains Malaysia, Malaysia). Knock-down was recorded every 15 min up to 1-h. After the 60-min exposure, mosquitoes were transferred back to the holding tubes and mortality was recorded after 24-h.

### PCR screening for *rdl*

Genomic DNA was extracted from 290 *An. arabiensis* SENN which underwent broflanilide bioassay testing and 219 *An. arabiensis* SENN which were exposed to dieldrin. Individual mosquitoes were homogenized in a Qiagen TissueLyser II (Qiagen, UK) with sterilized 5 mm stainless steel beads for 5 min at 30 Hz and incubated overnight at 56 °C. DNA was extracted using DNeasy® 96 Blood and Tissue Kits (Qiagen, UK), according to the manufacturer’s protocol.

Individual mosquitoes were identified to species-level using species-specific PCR primers for *An. gambiae s.s.* and *An. arabiensis* (Table 3)^63^. Each 20 μl reaction contained 20–40 ng of gDNA, 10 μl HotStart Taq 2X Master Mix (New England Biolabs, UK) and 25 pmol/ml of primers AR-3T, GA-3T and IMP-UN. Prepared reactions were run on a BioRad T100™ thermal cycler with the following conditions: 95 °C for 5 min, followed by 30 amplification cycles (95 °C for 30 s, 58 °C for 30 s, 72 °C for 30 s) and a final elongation step at 72 °C for 5 min. PCR products were visualised on 2% E-gel agarose gels in an Invitrogen E-gel iBase Real-Time Transilluminator. A Quick-Load® 100 bp DNA ladder (New England Biolabs, UK) was used to determine band size. PCR products of 387 bp or 463 bp were indicative of *An. arabiensis* or *An. gambiae s.s.*, respectively, relative to positive controls; no-template negative controls were included with all reaction runs.

**Table 3:** PCR primer and probe sequences.

PCR Assay	Primer/probe name	Primer/probe sequence
*An. gambiae* species identification	IMP-UN	GCTGCGAGTTGTAGAGATGCG
	GA-3T	GCTTACTGGTTTGGTCGGCATGT
	AR-3T	GTGTTAAGTGTCCTTCTCCGTC
A296S *rdl* detection	SerRdlF	TCATATCGTGGGTATCATTTTGGCTAAAT
	SerRdlR	TCGTTGACGACATCAGTGTTGT
	WT2	/HEX/TTACACCTA/ZEN/ATGCAACACG/3IABkFQ/
	Ser	/FAM/CACCTAATG/ZEN/AAACACG/3IABkFQ/

The presence of the A296S *rdl* mutation in *An. arabiensis* was determined using a TaqMan assay^70^. Each 20 μl reaction contained 20–40 ng of gDNA, 10 μl 2X PrimeTime® Gene Expression Master Mix (Integrated DNA Technologies, USA), 800 nM of primers SerRdlF and SerRdlR and 200 nM of probes WT2 and Ser (Table 3). Prepared reactions were run on a Stratagene Mx3005P qPCR system with the following conditions: 95 °C for 10 min, followed by 40 amplification cycles (95 °C for 10 s, 60 °C for 45 s), and lastly a dissociation curve. No-template negative controls were included with all reaction runs. The presence of a wild-type individual was indicated by a substantial increase in HEX signal, the presence of the A296S *rdl* mutation was indicated by a substantial increase in FAM signal; increase in both signals indicated a heterozygote.

### Data analysis

Discriminating concentration (DC) determination was undertaken using BioRssay^71^ in RStudio v4.0.2^72^. Mortality-dose regression analysis using a generalized linear model was performed per mosquito strain. Lethal doses for 50%, 95% and 99% (LC_50_, LC_95_ and LC_99_) with 95% confidence intervals were calculated. The LC_95_ value was multiplied by three to determine the DC as per the Lees et al*.* method^31^. The DC was also calculated by multiplying the LC_99_ by two as per the WHO approach^32^. Differences in dose-mortality responses between strains were evaluated using pair-wise comparisons with Bonferroni correction. All other statistical analyses were conducted in GraphPad Prism 9.4.0.

### Ethics approval

Ethical approval for the study was obtained from the London School of Hygiene and Tropical Medicine (LSHTM; ref#26035) and the National Institute for Medical Research (NIMR) in Tanzania (NIMR/HQ/R.8a/VOL.IX/3520). KCMUCo and NIMR obtained approval from the Animal Welfare and Ethical Review Board of LSHTM (ref#2019-14) for use of animals for mosquito maintenance. Study procedures and reporting are in accordance with the Animal Research: Reporting of In Vivo Experiments (ARRIVE) guidelines. All study procedures were performed in accordance with relevant guidelines and regulations.

## Data Availability

The datasets generated and/or analysed during the current study are available from the corresponding author upon reasonable request.
